# WormBase single-cell tools

**DOI:** 10.1093/bioadv/vbac018

**Published:** 2022-03-28

**Authors:** Eduardo da Veiga Beltrame, Valerio Arnaboldi, Paul W Sternberg

**Affiliations:** Division of Biology and Biological Engineering, Caltech, Pasadena, CA 91125, USA; Division of Biology and Biological Engineering, Caltech, Pasadena, CA 91125, USA; Division of Biology and Biological Engineering, Caltech, Pasadena, CA 91125, USA

## Abstract

We present two web apps for interactively performing common tasks with single-cell RNA sequencing data: *scdefg* for differential expression and *wormcells-viz* for visualization of gene expression. We deployed these tools with public *Caenorhabditis elegans* datasets curated by WormBase at https://single-cell.wormbase.org. Source code for deploying these tools with other datasets is available at https://github.com/WormBase/scdefg and https://github.com/WormBase/wormcells-viz.

**Supplementary information:**

Supplementary data are available at *Bioinformatics Advances* online.

## 1 Introduction

The number of single-cell RNA sequencing (scRNA-seq) publications has exploded in recent years, with over 1200 studies currently available and over 350 new studies in 2020 alone (Svensson *et al.*, 2020). This wealth of data is far from being used to its full potential, as most methods and tools for scRNA-seq data require programming proficiency in Python or R. Leveraging scRNA-seq data beyond the original studies demand tools that are easy to use and enable the integration, query and display of data in ways that are useful for scientists. Over 85% of scRNA-seq studies use human or mouse samples, and the volume of data generated for these organisms is so high that their integration and unified management presents a formidable challenge by itself. But for other organisms such as *Caenorhabditiselegans*, for which there are only on the order of a dozen studies in the literature (Table 1), data curation, integration and maintenance of tools encompassing most of the published studies is manageable by a single individual or research group using simpler tools.

**Table 1.** Number of scRNA-seq studies for the most popular organisms

Organism	Studies
Mouse	564
Human	482
Human and mouse	102
Zebrafish	28
*Drosophila*	14
Rat	12
*Caenorhabditis elegans*	8
Chicken	4
Yeast	3
*Arabidopsis thaliana*	3

*Note:* Data from Svensson et al. (2020; https://nxn.se/single-cell-studies).

In this spirit, we present two tools: *scdefg*, for performing interactive differential expression (DE), and *wormcells-viz*, for visualization of gene expression data. These tools leverage the *anndata* file format (Alexander Wolf *et al.*, 2018), a standard file format for scRNA-seq data and annotated data matrices and *scvi-tools* (Gayoso *et al.*, 2022), a popular framework for generative modeling of scRNA-seq data and statistical analysis (https://scvi-tools.org).

## 2 Overview of the *scdefg* and *wormcells-viz* tools

The *scdefg* app provides a single web page with an interface for performing DE on two groups of cells that can be selected according to the existing annotations in the data. For example, the user can select a group according to a combination of cell type, sample, tissue and experimental group. DE is computed using the scVI model (Lopez *et al.*, 2018) from scvi-tools (Gayoso *et al.*, 2022), which enables quick computation even when using only CPUs. The results are displayed in the form of an interactive volcano plot (log fold change versus *P*-value) and MA plot (log fold change versus mean expression) that display gene descriptions upon mouseover, and sortable tabular results that can be downloaded in csv and Excel format. The app is written in Python using Flask and Plotly, and can be launched from the command line by specifying the path to a trained scVI model, plus the data labels by which cell groups may be stratified (e.g. cell type, experiment and sample). We have deployed the app on a cloud instance with only 8 GB RAM and two vCPUs and observed this configuration is sufficient for handling a few concurrent users with results returned in about 15 s.

The *wormcells-viz* app provides interactive and responsive visualizations of heatmaps, gene expression histograms and swarm plots. It is written in Javascript and Python and uses React.js and D3.js. Deploying the app requires having precomputed gene expression values stored in three custom anndata files as described in the Supplementary Material. Using one anndata file for each visualization type keeps the codebase modular and simplifies adding more visualizations in the future. In the *wormcells-viz* documentation, we provide a pipeline and tutorial to compute these expression values using scVI with any scRNA-seq dataset. The pipeline could be adapted for using other scRNA-seq analysis frameworks, but our recommendation is to use scvi-tools because of considerations of scalability, speed of model training and consistent codebase development, as further discussed in the Supplementary Material. The following visualizations are currently implemented in *wormcells-viz*.

### 2.1 Heatmap

Visualization of mean gene expression in each group annotated in the data. The expression rates can be shown as either a traditional heatmap, or as a monochrome dotplot.

### 2.2 Gene expression histogram

Histograms of the gene expression rates for a given gene across all cell types in the data. The histogram bin counts are computed from the scVI inferred expression rates for each cell.

### 2.3 Swarm plot

This is a new visualization strategy to facilitate candidate marker gene identification. For a given cell type, swarm plots visualize the relative expression of a set of genes across all cells annotated in a dataset. The *Y*-axis displays the set of selected genes, and the *X*-axis displays the log fold change in gene expression between the cell type of interest and all other cell types. This is computed by doing pairwise DE of each annotated cell type versus the cell type of interest.

### 2.4 WormBase standard anndatas

In order to facilitate WormBase scRNA-seq data curation, we developed simple data wrangling guidelines for structuring anndata files with standard named fields. Having standardized data enables straightforward usage in software pipelines. Following these guidelines, we curated high-throughput scRNA-seq *C. elegans* datasets and made it available at Caltech Data (https://data.caltech.edu). See Supplementary Material for details on the curated datasets and the WormBase standard anndata convention.

### 2.5 Prospects for the alliance of genome resources

WormBase is a member of the Alliance of Genome Resources (https://alliancegenome.org), a consortium of model organism databases that encompasses zebrafish, *Drosophila melanogaster*, mice, rat and yeast. In this work, we have curated all of the available *C. elegans* data and made it available for the community. For other model organism databases, it is also feasible to manually curate all the relevant public datasets. Once the data are curated, integrating such massive aggregated datasets with scvi-tools becomes straightforward. By leveraging the tools presented here it is possible to offer users an interface to query and compare data from several studies in a way that is quick and useful but without the need to write code.

## Supplementary Material

vbac018_Supplementary_Data
