# Supplementary material for: WormBase single-cell tools
Source: Bioinform Adv. 2022 Mar 28;2(1):vbac018. doi: 10.1093/bioadv/vbac018 (PMC9258504; doi:10.1093/bioadv/vbac018)
Supplement: vbac018_Supplementary_Data [file vbac018_supplementary_data.pdf]

Supplementary Material
WormBase single cell tools

Eduardo da Veiga Beltrame, Valerio Arnaboldi & Paul W. Sternberg

{beltrame,valearna,pws}@caltech.edu

Division of Biology and Biological Engineering, Caltech

Pipeline Overview

Figure S1 provides a schematic overview of generating the assets needed to deploy *scdefg*, which only requires a trained scVI model, or *wormcells-viz*, which requires generating custom anndata files.

Code and data availability

- Web deployments with *C. elegans* scRNA-seq data: single-cell.wormbase.org
- Source code for *scdefg* and deployment instructions: github.com/WormBase/scdefg
- Source code for *wormcells-viz* and deployment instructions: github.com/WormBase/wormcells-viz.
- WormBase convention for anndata standard field names: github.com/WormBase/anndata-wrangling

Rationale for using scvi-tools

The scvi-tools framework offers several models for single cell omics data, and for scRNA-seq in particular offers the scVI model [1], which is bayesian hierachical generative model that leverages variational autoencoders to enable robust statistical analysis. It is built with PyTorch (pytorch.org) and has been extensively validated [2]. Training the scVI model only requires a gene count matrix, as outputted by scRNA-seq alignment software such as Cell Ranger [3]. There are currently hundreds of software tools and pipelines developed for scRNA-seq data [4], and below we outline the considerations that led to our choice of using scvi-tools.

Scalability: scvi-tools models readily scale datasets with millions of cells. Using a GPU even large models for millions of cells can be trained in a few hours, and models for a few thousand cells are trained in minutes. New datasets can also be integrated to an existing model without re-training from scratch.

Consistent Development and Contributors: The scvi-tools codebase (https://github.com/scverse/scvi-tools) was first introduced in 2017, and published in 2018 [1], with consistent updates and improvements since then [2]. It now boasts a mature and professional API and codebase that follows industry best practices, and has over 45 unique contributors and 43 releases.

Extensible Framework for Analysis: Because the generative model of the data can be modified to capture our assumptions about underlying processes, the framework can be extended to model other aspects of scRNA-seq data. Currently, extensions include cell type classification and label transfer across batches, modelling single cell protein measurements, single cell chromatin accessibility assays, gene imputation in spatial data, and using a linear decoder to allow for interpretation of the learned latent space. Several peer reviewed articles have been published describing these extensions (see scvi-tools.org/press).

WormBase deployment rationale

At the moment, the majority of scRNA-seq studies generate data using the 10X Genomics Chromium technology [5]. This is also true for *C. elegans* scRNA-seq data. For the time being WormBase will focus development efforts on scRNA-seq tools on 10X Genomics data. Two considerations drive this:

i) Data integration of different batches with scvi-tools is more robust when there is more data, and when the technology and biological system of each batch is the same or similar. Attempting to integrate a small number of cells from unique technologies and unique biological systems can make it impossible to discern biological differences from technical artifacts.

ii) 10X Genomics supports the Cell Ranger [3] software pipeline for going from FASTQ files to gene count matrices. Technology standardization enables WormBase to uniformly reprocess FASTQ files in a single pipeline in the future.

WormBase standard anndata convention

The Anndata file format (extension type `.h5ad`) was published in 2018 [6] as a generic class for handling annotated data matrices, with a focus on scRNA-seq data and Python support for machine learning, and integration with the SCANPY analysis framework (scanpy.readthedocs.io). Anndata is an efficient storage format because it uses HDF5 compression, and has come to be the standard format for manipulating scRNA-seq data in Python, as well as providing support in R (see github.com/theislab/zellkonverter and cran.r-project.org/web/packages/anndata/index.html) .

Anndata's popularity and uses continues to grow, with many packages standardizing their data maniulation around it. Examples include scvi-tools (scvi-tools.org), the Chan Zuckerberg cellxgene platform (chanzuckerberg.github.io/cellxgene) and the COVID-19 cell atlas initiative which standardized data distribution around anndata (covid19cellatlas.org). Owing to the advantages of anndata and its popularity, WormBase adopted simple data wrangling guidelines for structuring published scRNA-seq data into anndata files with standard field names, in order to streamline their reuse in code pipelines. These guidelines are described in tables S1 and S2 and maintained at github.com/WormBase/anndata-wrangling.

Related work

Prior to building these tools we searched the scRNA-tools database [4] (https://scrna-tools.org/) for existing Python tools that could be extended with the functionalities we envisioned. The CZI cellxgene tool (https://cellxgene.cziscience.com/) was the only one deemed sufficiently robust to being extended. However due to the large software complexity, for development and maintainability it was deemed preferable for WormBase to have independent, smaller, standalone tools.

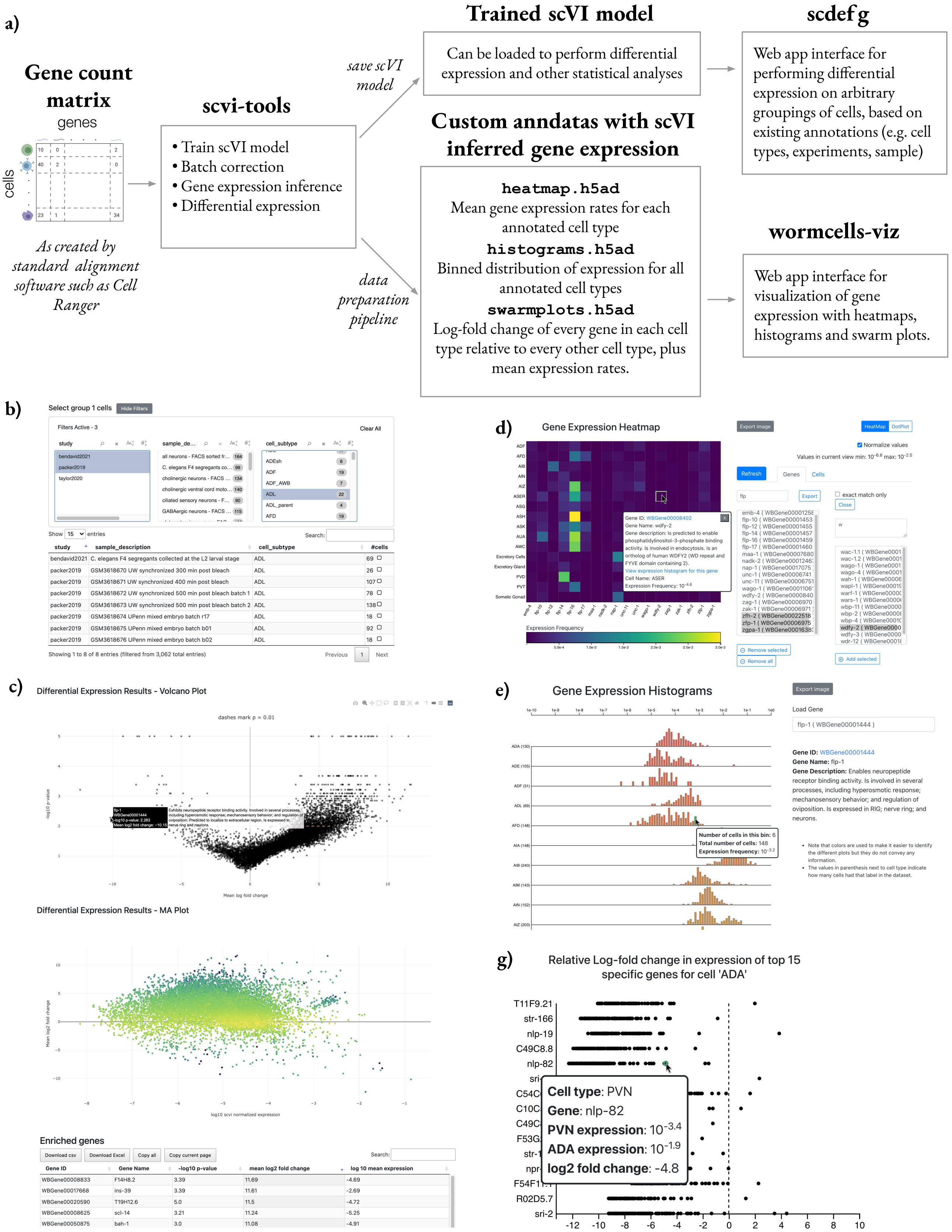

Figure S1: The WormBase single cell tools. a) Overview of the process to go from gene count matrix to deployment of the apps. Training a scVI model can be done quickly and only requires starting from the gene count matrix as outputted by standard alignment software such as Cell Ranger [3]. The *scdefg* app only requires as input the trained scVI model as saved by scvi-tools, while *wormcells-viz* requires using our pipeline to create the custom input anndatas, which are saved as `.h5ad` files (see supplements). **b)** The cell selection filter interface of *scdefg*. **c)** Results showing volcano plot, MA plot and part of the tabular results view of *scdefg* . **d)** Heatmap of *wormcells-viz*. **e)** Gene expression histogram of *wormcells-viz*, showing scVI normalized expression rates. **f)** Swarm plot of *wormcells-viz*.

Description of *wormcells-viz* custom anndatas

In this section we briefly review the conventions of the format expected by *wormcells-viz* for an anndata object instantiated in Python as `adata = anndata.AnnData`. A Jupyter notebook tutorial, and a Python script that goes from gene count matrices to the anndata files are described in the repository documentation at github.com/WormBase/wormcells-viz

Gene expression histograms anndata formatting

These are used for plotting histograms of the scVI inferred expression rates for a given gene across all cell types in the data. Each anndata layer stores the expression values for all cell types and a given gene. The histogram bin counts are computed from the scVI normalized expression values, which are generated by a trained scVI model using the method `scvi.model.SCVI.get_normalized_expression`. The anndata `obs` contains the cell types and `var` contains the histogram bins, the genes are stored in layers with the keys being the gene ID. We store the genes in the layers because each view in the *wormcells-viz* app show the histograms for a single gene, so this makes accessing the data simpler. The histogram bin counts are computed from the $\log_{10}$ of the scVI expression rate. Each bin contains the number of cells in the dataset that were inferred to have an expression rate in the bin interval. There should be 100 bins with values between $(-10, 0)$, representing expression rates from 10^{-10} to 10^{0}. The data array is of shape $n_{celltypes} \times n_{bins} \times n_{genes}$. The `adata` properties should be:

- `adata.obs` := Dataframe with cell types in index.
- `adata.var` := Dataframe with the bin intervals in index.
- `adata.X` := Not used.
- `adata.layers[gene_id]` := Each layer key is a gene ID, and contains a matrix with the binned expression rate counts for all cell types.
- `adata.uns['about']` := String with dataset information.

Heatmap anndata formatting

These are used for plotting a heatmap of the average expression rates in each cell type, for a given selection of cell types and genes. The input data is a matrix of cell types and gene expression rates that contains the $\log_{10}$ scVI expression rate values. Cell types are in `adata.obs` and genes in `adata.var`. The data array is of shape $n_{celltypes} \times n_{genes}$. The `adata` properties should be:

- `adata.obs` := Dataframe with cell types in index.
- `adata.var` := Dataframe with gene IDs in index.
- `adata.X` := Matrix with $\log_{10}$ values of scVI normalized expression for each cell type and each gene.
- `adata.uns['about']` := String with dataset information.

Swarm plots anndata formatting

These are used for plotting relative expression of a set of genes across all cells annotated in the dataset. The Y axis displays the set of selected genes, and X axis displays the log fold change of expression of that gene on all cell types relative to the cell of interest. The fold change is computed by doing pairwise differential expression of each annotated cell type vs the cell type of interest. For a given cell type, genes can be sorted by p-value, log fold change or mean expression rate. This part of the data is an array of shape $n_{celltypes} \times n_{genes} \times n_{celltypes}$. The cell types are repeated along two dimensions, because this data contains the results of pairwise DE comparisons among each cell type in the data.

Plus $n_{celltypes}$ matrices shaped like $n_{celltypes} \times n_{genes}$, because each unstructured layer `adata.uns[celltype]` contains a dataframe with global differential expression results for that cell type.

Finally, the unstructured layer `anndata.uns['heatmap']` contains a matrix with $\log_{10}$ scVI expression rates heatmap data (same data as used for plotting the heatmap), with genes in the index and cell types in the columns. This data is used to display the expression of each cell type on mouseover. The `adata` object properties should be:

- `adata.obs` := Dataframe with cell types in index.
- `adata.var` := Dataframe with gene IDs in index.
- `adata.X` := Not used.
- `adata.layers[cell_type]` := Mean log fold change for a given cell type for all genes.
- `adata.uns[cell_type]` :=The differential expression tables of the corresponding cell type vs all other cells. This can be used for ordering the genes by p-value, log fold change, and expression rate.
- `adata.uns['heatmap']` := Dataframe with genes in index and cell types in columns containing the $\log_{10}$ of the scVI expression frequency for each cell type
- `adata.uns['about']` := String with dataset information.

References

[1] Lopez et al. (2018): *Deep generative modeling for single-cell transcriptomics*, Nature Methods: 10.1038/s41592-018-0229-2.
[2] Gayoso et al. (2021): *scvi-tools: a library for deep probabilistic analysis of single-cell omics data*, bioRxiv: 10.1101/2021.04.28.441833.
[3] Zheng et al. (2017): *Massively parallel digital transcriptional profiling of single cells*, Nat. Commun.: 10.1038/ncomms14049.
[4] Zappia et al. (2018): *Exploring the single-cell RNA-seq analysis landscape with the scRNA-tools database*, PLOS Comp. Biol.: 10.1371/journal.pcbi.1006245.
[5] Svensson et al. (2020): *A curated database reveals trends in single-cell transcriptomics*, Database: 10.1093/database/baaa073.
[6] Wolf et al. (2018): *SCANPY: large-scale single-cell gene expression data analysis*, Genome Biology: 10.1186/s13059-017-1382-0.
[7] Hashimshony et al. (2012): *CEL-Seq: single-cell RNA-Seq by multiplexed linear amplification*, Cell Reports: 10.1016/j.celrep.2012.08.003.
[8] Tintori et al. (2016): *A Transcriptional Lineage of the Early C. elegans Embryo*, Developmental Cell: 10.1038/10.1016/j.devcel.2016.07.025.
[9] Cao et al. (2017): *Comprehensive single-cell transcriptional profiling of a multicellular organism*, Science: 10.1126/science.aam8940.
[10] Packer et al. (2019): *A lineage-resolved molecular atlas of C. elegans embryogenesis at single-cell resolution*, Science: 10.1126/science.aax1971.
[11] Taylor, Seth R et al. (2020): *Molecular topography of an entire nervous system*, bioRxiv: 10.1101/2020.12.15.422897.
[12] Ben-David et al. (2021): *Whole-organism eQTL mapping at cellular resolution with single-cell sequencing*, Elife: 10.7554/eLife.65857.

Table S1: WormBase naming guidelines for the anndata `var` annotation names - `anndata.AnnData.var`
https://anndata.readthedocs.io/en/latest/anndata.AnnData.var.html

`var` name	Description	Example value	Optionality
**`index`**	WormBase gene ID	WBGene00010957	Required
**`gene_id`**	WormBase gene ID	WBGene00010957	Required
**`gene_name`**	WormBase gene name	nduo-6	Required
**`gene_description`**	WormBase short gene description.	Predicted to have NADH dehydrogenase activity.	Optional

Table S2: WormBase naming guidelines for the anndata `obs` annotation names - `anndata.AnnData.obs`
https://anndata.readthedocs.io/en/latest/anndata.AnnData.obs.html

`obs` name	Description	Example value	Optionality
**`index`**	The batch name joined with cell barcode witha + char	F4_1+TGTAACGGTTAGCTAC-1	Required
**`study`**	A unique shorthand for the study that published the data, ideally in the style all lower case.	bendavid2021	Required
**`sample_batch`**	The run that produced the corresponding barcode. Typically **`sample_batch`** and **`sample`** will be the same, but with multiplexing one batch can have multiple samples	F4_1	Required
**`sample`**	The name of the biological sample that is in this batch	L2 larvae batch 4	Required
**`sample_description`**	Description of the sample. This is mandatory because otherwise it would be easy to confuse two samples from their short name.	F4_1	Required
**`barcode`**	The cell barcode	AAACCCAAGATCGCTT-1	Required
**`cell_type`**	The cell type annotation provided by the authors. The value should be the string **unlabeled** if not available.	Neuronal	Required
**`cell_subtype`**	The lowest level of cell type annotation if provided by the authors. If only one level of cell type label was provided, it should be repeated in both **`cell_type`** and **`cell_subtype`**. This value should be the string **`unlabeled`**	ASJ	Required

Table S3: Summary of *C. elegans* single cell RNA sequencing datasets. High throughput data has been wrangled following the WormBase standard anndata convention and deposited at CaltechData (data.caltech.edu).

Author and year	Ref	Accession	CaltechData DOI	Description
Hashimshony 2012	[7]	SRP014672	Not wrangled	This was one of the pioneering works in scRNA-seq and introduced the CEL-Seq technique. They reported 96 *C. elegans* cells.
Tintori 2016	[8]	GSE77944	Not wrangled	They surveyed the *C. elegans* embryo through the 16-cell stage and reported 216 cells. They made a custom visualizer at tintori.bio.unc.edu.
Cao 2017	[9]	GSE98561	10.22002/D1.2000	The first high throughput scRNA-seq study on *C. elegans*, they introduced the sci-RNA-seq technique and surveyed over 50,000 cells from whole organism L2 larvae.
Packer 2019	[10]	GSE126954	10.22002/D1.1945	They performed a comprehensive survey of the *C. elegans* embryo developmental trajectory with over 86,000 cells with 10X Genomics v2 chemistry. They made a custom visualizer at cello.shinyapps.io/celegans
Taylor 2020	[11]	GSE136049	10.22002/D1.1977	As part of the CeNGEN project (cengen.org) they FACS sorted L4 larvae neurons and surveyed over 101,000 cells using 10X Genomics v2 and v3 chemistry. They report 65,000 neurons across all neuron types. They made a custom visualizer at cengen.shinyapps.io/CengenApp.
Ben-David 2021	[12]	PRJNA658829	10.22002/D1.1972	They surveyed over 55,000 cells of L2 larvae using 10X Genomics v2 chemistry.
